# Unusual histopathological findings in appendectomy specimens with clinical diagnosis of acute appendicitis: A retrospective cohort analysis

**DOI:** 10.1016/j.amsu.2021.102720

**Published:** 2021-08-21

**Authors:** Hamzeh Al-Balas, Raith S. Al-Saffar, Mahmoud Al-Balas, Mohammad K.M. Al-Wiswasy, Ala'a Abu Salhiyeh, Yasmeen Al-Sharqi, Mustafa Saad Yousuf, Kamal Bani-Hani

**Affiliations:** a Department of General and Special Surgery, Faculty of Medicine, Hashemite University, Zarqa, 13133, Jordan; b Department of Basic Medical Sciences, Faculty of Medicine, Hashemite University, Zarqa, 13133, Jordan; c Jordan University Hospital, Amman, Jordan; d Department of Histopathology, Prince Hamza Teaching Hospital, Amman, Jordan

**Keywords:** Acute appendicitis, Unusual findings, Serositis, Enterobius vermicularis, Appendiceal neoplasms

## Abstract

**Introduction:**

While appendicitis is considered one of most common acute surgical conditions, several studies have reported abnormal histopathological findings in appendectomy specimens; however, sending all appendices to histopathology is not yet routinely done.Here we report many unusual findings. Those unusual findings played a role not only in confirming acute appendicitis as a cause of the presentation in some cases but also discovering etiologies that mimic it with great impact on its management.

**Methods:**

Between January 2011 and December 2017, a total of 1510 patients were operated with appendectomy for a primary diagnosis of acute appendicitis. Among them, a total of 72 patients had incidental histopathologic findings in association with acute appendicitis or other pathologies instead of acute appendicitis. A retrospective analysis for those 72 patients was performed with all data being retrieved from the electronic health record system.

**Results:**

Patients ages ranged between 4 and 71 years with a mean age equal to 23.1 years (SD = 14.2). Majority of patients were women (n = 52; 72.2%). Sixty of the seventy-two cases were seen in patients with negative appendectomies (n = 333) with an overall rate of 18% among this group of patients. The remaining 12 patients had additional findings in histopathology specimens beside acute appendicitis (n = 1131) with an overall rate of 1%. The most commonly reported pathologies were serositis, ovarian cysts, and Enterobius vermicularis in descending frequency.

**Conclusion:**

Identification of unusual histopathological findings during microscopic examination of resected appendices is more common in female patients and in patients with negative appendectomy. histopathologic assessment of specimens will allow detection of congenital, infectious or malignant pathologies that mimic acute appendicitis clinically even in the absence appendicitis microscopically.

## Highlights

• Appendicitis is a global disease, and one of the most common major general surgical emergencies.
• Identification of unusual histopathological findings is more common in female patients and in patients with negative appendectomy.
• Appendicitis can be secondary to different bacterial, viral, parasitic and malignant pathologies.
• critical histopathologic assessment of resected appendicies is mandatory to exclude secondary rare pathologies.

## 1 Introduction

Appendicitis is a global disease, and it's without doubt one of the most common major general surgical emergencies [1,2]. Men and women in the United States have approximately 1 in 12 and 1 in 15 lifetime risks of getting appendicitis, respectively.

Obstruction of the appendiceal lumen by various causes seems to be the most likely origin of appendicitis [3]. Despite the fact that the obstruction resulted usually from lymphoid hyperplasia secondary to inflammatory bowel disease or infections, fecal stasis and fecaliths, It has been less commonly attributed to various unusual causes like bacteria (Yersinia species [4], adenovirus [5], cytomegalovirus, actinomycosis [4], Mycobacteria species, Histoplasma species), parasites [3,6] (e.g., Schistosomes species [3,5,7], Entamoeba histolytica [8,9]), Pinworms 'Enterobius vermicularis' [[10], [11], [12], [13]], enterocele [14], Ascariasis [11,15,16], serositis [10], Eosinophilic infiltration [5,7,11], foreign bodies [4,17], tuberculosis [4,7,[18], [19], [20]], carcinoid [3,7,11,[21], [22], [23], [24]], mucocele [11,16], Endometriosis [5,[25], [26], [27], [28], [29]],Intussusception [17,29,30] Diverticulosis and Diverticulitis [17,[31], [32], [33]], or even malignancy such as adenocarcinoma [3,5,11,24,25] or appendiceal neuroma [33,34].This paper aims to identify different patterns of unusual histopathological findings in appendiceal specimens in patients with clinical diagnosis of acute appendicitis as well as their prevalence.

## 2 Materials and methods

Registration and ethics: Research Registry number is stated, in accordance with the declaration of Helsinki. Unique identifying number: researchregistry6963 (https://www.researchregistry.com/browse-the-registry#home/registrationdetails/60ea0c7a7f4cbf00210ee916/)

Ethical approval: The study was approved by the Institutional Review Board (IRB) in Hashemite University.

This is a retrospective cohort analysis of the histopathologic findings of patients who were operated with appendectomy for a provisional diagnosis of acute appendicitis in the period between January 2011 and December 2017 in prince Hamza hospital in Amman, Jordan (i.e., a tertiary referral governmental hospital affiliated with the Hashemite University College of Medicine). Patients who went through incidental appendectomy during other surgeries were excluded from the study. A total of 1510 patients was included in the study, 841 (56%) men and 669 (44%) women. Among them, a total of 72 patients proved to have unusual histopathological findings or other pathologic findings other than acute appendicitis.

A critical review of all medical records was performed and all patients' specimens labeled with abnormal findings other than acute appendicitis were reviewed by two experienced pathologists each of them with more than 30 years' experience in the field of pathology.

The main aim of the study is to identify different patterns of unusual histopathological findings in patients with provisional diagnosis of acute appendicitis and to assess their prevalence as well as their clinical significance.

This paper is prepared in compliance with STROCSS 2019 criteria [63]. Data were recorded in a Microsoft Excel (Redmond, WA, USA) spreadsheet and analyzed by SPSS program version 16.0. Statistical significance was assessed using a two-tailed Fisher's exact test (statistical significance was considered for p < 0.05).

## 3 Results

Unusual histopathological pathologic findings or other pathologic conditions other than acute appendicitis were found in 72 patients out of 1510 patients with appendectomy for a provisional diagnosis of appendicitis with an overall rate of 4.76%. Out of the seventy-two, twenty patients (27.8%) were males, and fifty-two (72.2%) were females. Patients ages ranged between 4 and 71 years, with a mean of 23.1 years (SD = 14.2).

Sixty of the seventy-two cases were seen in patients with negative appendectomies (n = 333) with an overall rate of 18% among this group of patients. The remaining 12 patients had additional findings in histopathology specimens beside acute appendicitis (n = 1131) with an overall rate of 1%.

The unusual histopathologic and uncommon findings seen in the seventy-two patients in descending frequency were serositis (n = 18), ovarian cyst (n = 16) among them 10 cases were hemorrhagic cysts, Enterobius vermicularis (n = 12), carcinoid tumor (n = 4), small intestine infarction (n = 3), Entamoeba histolytica (n = 2), single cases of primary adenocarcinoma, ruptured ectopic pregnancy, endometriosis, ovarian dermoid cyst, ovarian endometriotic cyst, para fallopian tube cyst, foreign body granuloma, cecal abscess, transmural infarction of the caecum, Meckel's perforated diverticulitis with peritonitis and abscess, Eosinophilic infiltration, Intussusception, Crohn's disease, Neuroma of appendicular tip, Hyperplastic mucinous polyp, Appendicular diverticulum and Cecal fistula. The clinicopathological characteristics of the 72 cases are summarized in Table 1.

**Table 1:** **Patients with unusual histopathologic/other pathologic conditions (n = 72)**.

Histopathologic finding	Number	Female/Male	Acute Appendicitis
Serositis	18	F: 12 (6,11,11,23,26,27,31,32,34,35,37,69years) M: 6 (6,8,27,27,32,36 years)	0
Ovarian cyst	16	F: 16 (14,14,15,15,16,17,17,18,19,22,23,24,27,38,43,48 years)	2
Oxyuris (Entrobius Vermicularis)	12	F: 9 (10,21,10,6,15,17,19,17,12 years) M: 3 (4,9,19 years)	3
Carcinoid tumor	4	F: 3 (16,17,28 years) M: 1 (27 year)	1
Transmural infarction of small intestine	3	M: 3 (28,32,71 years)	2
Entameba Histolytica	2	M: 2 (16 and 17 years)	0
Primary adenocarcinoma of the distal appendicular lumen with lymph node secondaries	1	F (39 year)	0
Ruptured ectopic pregnancy	1	F (34 year)	0
Endometriosis	1	F (24 year)	0
Ovarian dermoid cyst	1	F (35 year)	0
Ovarian endometriotic (Chocolate) cyst	1	F (23 year)	0
Para fallopian tube cyst	1	F (27 year)	0
Foreign body granuloma	1	F (34 year)	0
Cecal abscess	1	F (20 year)	0
Transmural infarction of caecum	1	F (63 year)	0
Perforated Meckel's diverticulitis with peritonitis and abscess	1	M (54 year)	0
Eosinophilic infiltration	1	M (17 year)	0
Intussusception	1	M (9 year)	0
Crohn's disease	1	M (21 year)	0
Neuroma of appendicular tip	1	M (28 year)	1
Hyperplastic mucinous polyp	1	M (11 year)	1
Appendicular diverticulum (Not inflamed)	1	F (54 year)	1
Cecal fistula	1	F (11 year)	1

## 4 Discussion

Appendicitis is the most common surgical emergency that mostly affects adolescents although it can hit any age [35]. The primary pathology is luminal obstruction which increases the intra-luminal pressure within the appendix, and leads to ischemia. Bacteria translocate causing inflammation, Infarction and perforation can happen after [36]. Many usual and unusual etiologies may lead to appendiceal lumen obstruction. An overview of reported histopathological findings is discussed in our paper.

### 4.1 Serositis

Also called peri-appendicitis, is an inflammation of the serosal surface of the appendix, which is always associated with an intra-abdominal pathology [37]. It is difficult to be diagnosed clinically. The disease course depends on early recognition and treatment of the underlying cause. among our patients, it was the most reported histopathology findings in patients with negative appendectomy, and it was more prevalent among women. Among all patient who had appendectomy (n = 1510), 18 patients (12 females, 6 males) with mean age of 26.5 and age range 6–69 years had a diagnosis of serositis without evidence of underlying appendicitis. Jadhav V and Singhal V [10] reported 5 patients who had serositis in 199 appendices. Peri-appendicitis actually has been found in many other studies [38,39].

### 4.2 Ruptured ovarian corpus luteal cyst

Most of ruptured ovarian cysts could be asymptomatic or even present with minimal symptoms mimicking acute appendicitis, which can be controlled medically. in some cases, it may develop complications and require surgical intervention.

Among the 72 patients, 16 female patients with mean age of 23.1 years and age range 11–48 years were diagnosed with ruptured ovarian cyst during appendectomy with an overall rate of 1.05%. only 2 patients had associated acute appendicitis, 10 of the cysts were hemorrhagic and 6 were simple, non-hemorrhagic corpus luteal cysts. Although the association of a ruptured ovarian cyst and acute appendicitis is unlikely but it can happen. Tanaka [40] reported three patients who showed ruptured ovarian cysts in association with acute appendicitis.

### 4.3 Enterobius vermicularis

Formerly known as Oxyuris vermicularis is an extremely common among population, which runs in families. It is commonly asymptomatic with high cure rate but common recurrences.A total of 12 patients including 9 females and 3 males were diagnosed with E. vermicularis on the final histopathology for appendectomy with an overall prevalence of 0.79%. Patients ages range from 4 to 21 years, with a mean of 13 years. 3 patients (2 females and one male patients) had E. vermicularis with acute appendicitis. its association with appendicitis was first reported in the late 19 ^th^ century. Previous reports of E. vermicularis incidence in appendectomy specimens have ranged from 0.2% to 41.8%. In this study the incidence (i.e., 0.79%) was very close to 0.81% reported by Yabanoglu [9] (12/1466) and 0.6% by Emre (2013) [57]. Qasaimeh et al. [61] reported a prevalence of 2.2% in their study on 3984 appendices in northern Jordan. Rates of inflammation in appendices infected with E. vermicularis were ranged from 13% to 37% [5], in the present study it was 25% (3 out of 12 case) that is similar to other published studies.

### 4.4 Carcinoid tumor

The little understood slow-motion cancer is the most common tumor of the appendix. Appendix is the site of around 12% of carcinoid tumors, and it usually discovered as an incidental finding of histopathological examination following appendectomy.

Since the misdiagnosis of its symptoms, carcinoid tumor is usually diagnosed biochemically or histologically. Although it can behave aggressively, localized carcinoids have excellent prognosis with a 5-year survival rate of 98% [43].

Appendiceal carcinoid was diagnosed in 4 patients; three females (16, 17 and 28 years old) and one male (27 years). only male patient had a diagnosis of acute appendicitis in association with the carcinoid while all women had no histopathological evidence of appendicitis. All carcinoid tumors were localized in the distal part of the appendix, of which 3 cases were approximately 4 mm in diameter while the 4th one reached 10 mm all tumors were of the insular type, and were invading muscularis propria without involvement of the serosa.

Literature review by Shrestha (2012) [62] showed incidence of carcinoid tumor ranging from 0.1% to 1.05%, mostly found incidentally during microscopic examination. In the present study, the incidence was 0.26% which lies within the range of other reports [62].

### 4.5 Transmural infarction

Any serious infection within the peritoneum can progress to intestinal infarction which is a life-threatening condition, therefore, early diagnosis and therapy is a must and occasionally surgery is essential. many complications of acute appendicitis occur even after appendectomy. in particular, thrombophlebitis of the porto-mesenteric veins could occur and progress to intestinal infarction. this is usually uncommon with the antibiotic use and the surgical management; however, it should always be considered [44].Transmural infarction was found in 3 males, 28 y old with negative appendectomy and two 32 y and 71 y old males with acute appendicitis.

### 4.6 Entameba Histolytica

Even it is rare to be a cause of acute appendicitis, E. histolytica must be kept in physician's mind to avoid misdiagnosis and unfavorable prognosis. It is diagnosed by histopathological examination with PAS stain postoperatively and a fecal culture. The treatment is usually appendectomy with oral metronidazole [45]. E. histolytica was seen in two young male patients (16 and 17-year-old), in both the appendix was not inflamed. Yabanoglu et al. [9] reported 4 cases of Entameba Histolytica in 1466 appendices, one of which was associated with acute appendicitis.

### 4.7 Primary adenocarcinoma (PAA)

PAA is very rare tumor, first described in 1882 with fewer than 300 cases recorded between 1882 and 2004 [3]. One case of primary adenocarcinoma of the appendix (PAA) of the colonic type was reported in the distal appendix of a 39 years old female patient with evident direct continuity of the carcinoma with the normal appendicular mucosa confirming that this is PAA and not arising from the caecum, with no evidence of acute appendicitis. This case represents an incidence of 1 in 1510 cases (0.06%) which lie within the range reported by others (0.01% in Miguel Leon Arellano 2016 [46] and 0.08% in Collins (1955) [47,48]. Elective treatment of right hemicolectomy was carried out for the patient few days after the initial diagnosis.

### 4.8 Ruptured ectopic pregnancy

One 34-year-old female patient had ruptured ectopic pregnancy at time of appendectomy that was proved negative for AA. Although it's rare for ectopic pregnancy and acute appendicitis to occur at the same time, there few cases were reported about simultaneous AA with ruptured ectopic pregnancy [[41], [49]].

### 4.9 Endometriosis

Appendiceal endometriosis, which has 2.8% prevalence in patients with endometriosis [50] has assimilated symptoms to acute appendicitis. Because of endometrial tissue response to hormonal changes, symptoms of appendiceal endometriosis overlap with menstrual cycle. In one case, appendiceal endometriosis was reported in a 24 years old female patient.

### 4.10 Miscellaneous findings

**Fig. 1:**
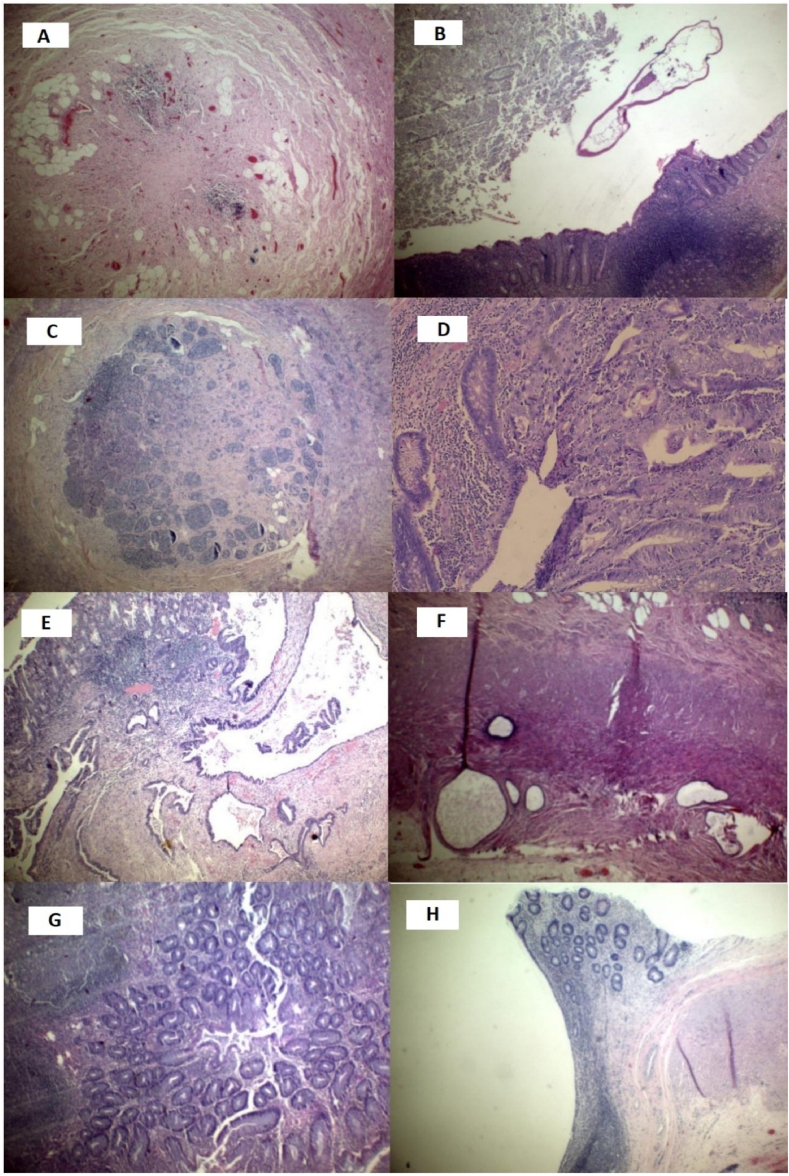
Unusual histopathological findings in the appendix (sections stained with hematoxylin and eosin (H&E). (A) Fibrous obliteration of the lumen, (B) E. vermicularis. Cross section of E. vermicularis in the appendix lumen, (C) Carcinoid tumor showing islands of carcinoid tumor cells in the central part of the appendix, (D) Adenocarcinoma with direct continuity of the carcinomatous cells with the normal appendicular mucosa indicating its primary origin, (E) The adenocarcinoma infiltrating the full muscular wall of the appendix, (F) Appendiceal endometriosis. Focus of endometriosis-containing endometrial glands and stroma in the appendicular subserosa, (G) Hyperplastic polyp of the appendiceal mucosa, (H) Appendiceal diverticulum showing pyloric glands metaplasia.

Other miscellaneous findings of variable clinical significance were reported in our group of patients as well as in the literature include Ovarian Chocolate cyst (i.e. endometrioma) [[42], [51]], para fallopian tube cysts [52], foreign body granuloma [53]., cecal abscess [54], transmural infarction of caecum [55], Meckel's diverticulum [56], eosinophilic infiltration [5,11,57]., intussusception [58], Crohn's disease of the appendix [59], Neuroma of appendix [60], hyperplastic polyps of appendix, cecal fistula and appendicular diverticulum [57]. Fig. 1 shows various unusual histopathological patterns in appendectomy specimens for different patients.

### 4.11 Strength and limitations

To the best of our knowledge, this is the first study that describes specifically unusual patterns of histopathological findings in patients with a presumptive diagnosis of acute appendicitis in Jordan. It has been performed in a tertiary hospital where large number of cases were retrospectively critically reviewed to confirm these unusual findings. Our findings were largely consistent with many reports from other world countries.

## 5 Conclusion

Identification of unusual histopathological findings during microscopic examination of resected appendices is more common in female patients and in patients with negative appendectomy. taking in consideration cases presented here in as well as in the literature, histopathologic assessment of specimens will allow detection of congenital, infectious or malignant pathologies that mimic acute appendicitis clinically even in the absence appendicitis microscopically. The clinical significance of identifying these unusual findings will impact the clinical outcome of affected patients for example by requiring further surgical interventions, adding chemotherapy for malignant changes or adding anthelmintic treatment.

## Funding statement

The Authors received no financial support for the research, authorship and/or publication of this article.

## Ethical approval

The statement of ethical approval was obtained from the Institutional Review Board (IRB) committee at Hashemite University AND Prince Hamza Hospital.

## Data availability

The data that support the findings of this study are available from the corresponding author upon reasonable request.

## Research registration number

Unique Identifying number or registration ID: researchregistry6963.

## Guarantor

The corresponding author is the guarantor for the work and he has the responsibility of access to the data, and controlling the decision to publish.

## Provenance and peer review

Not commissioned, externally peer-reviewed.

## Consent

Consent waived by the IRB.

## Author contribution

Hamzeh Al-Balas: Conceptualization, Methodology, Writing - original draft, Writing - review & editing, Raith S. Al-Saffar: literature review, Writing - review & editing, Mohammad K.M. Al-Wiswasy: Conceptualization, Data curation, Review of Specimens, Ala'a Abu Salhiyeh: literature review, Writing – original draft, Mahmoud Al-Balas: Conceptualization, Methodology, Writing - original draft, literature review, Yasmeen Al-Sharqi: Data curation, Review of Specimens, Kamal Bani Hani: Writing - review & editing.

## Declaration of competing interest

The authors declare no potential conflicts of interest with respect to the research, authorship and/or publication of this research.
